# Tunable Colossal Anomalous Hall Conductivity in Half‐Metallic Material Induced by *d*‐Wave‐Like Spin‐Orbit Gap

**DOI:** 10.1002/advs.202307288

**Published:** 2024-03-21

**Authors:** Joonyoung Choi, Jin‐Hong Park, Wonshik Kyung, Younsik Kim, Mi Kyung Kim, Junyoung Kwon, Changyoung Kim, Jun‐Won Rhim, Se Young Park, Younjung Jo

**Affiliations:** ^1^ Department of Physics Kyungpook National University Daegu 41566 South Korea; ^2^ Research Center for Novel Epitaxial Quantum Architectures Department of Physics Seoul National University Seoul 08826 South Korea; ^3^ Center for Correlated Electron Systems Institute for Basic Science Seoul 08826 South Korea; ^4^ Department of Physics and Astronomy Seoul National University Seoul 08826 South Korea; ^5^ Department of Physics Yonsei University Seoul 03722 South Korea; ^6^ Department of Physics Pohang University of Science and Technology Pohang 37673 South Korea; ^7^ Department of Physics Ajou University Suwon 16499 South Korea; ^8^ Department of Physics and Origin of Matter and Evolution of Galaxies (OMEG) Institute Soongsil University Seoul 06978 South Korea; ^9^ Integrative Institute of Basic Sciences Soongsil University Seoul 06978 South Korea

**Keywords:** anomalous hall effect, berry curvature, half‐metals

## Abstract

The anomalous Hall conductivity (AHC) in magnetic materials, resulting from inverted band topology, has emerged as a key adjustable function in spin‐torque devices and advanced magnetic sensors. Among systems with near‐half‐metallicity and broken time‐reversal symmetry, cobalt disulfide (CoS_2_) has proven to be a material capable of significantly enhancing its AHC. In this study, the AHC of CoS_2_ is empirically assessed by manipulating the chemical potential through Fe‐ (hole) and Ni‐ (electron) doping. The primary mechanism underlying the colossal AHC is identified through the application of density functional theory and tight‐binding analyses. The main source of this substantial AHC is traced to four spin‐polarized massive Dirac dispersions in the *k*_*z*_ = 0 plane of the Brillouin zone, located slightly below the Fermi level. In Co_0.95_Fe_0.05_S_2_, the AHC, which is directly proportional to the momentum‐space integral of the Berry curvature (BC), reached a record‐breaking value of 2507 Ω^−1^cm^−1^. This is because the BCs of the four Dirac dispersions all exhibit the same sign, a consequence of the *d*‐wave‐like spin‐orbit coupling among spin‐polarized *e*_*g*_ orbitals.

## Graphical abstract

This study presents a record‐breaking AHC observed in doped CoS_2_ in which the *d*‐wave orbital mixing is identified by spin‐orbit coupling as the key mechanism inducing large AHC, not around the Weyl points previously reported in CoS_2_. The unique mechanism proposed, leading to the phenomenally high conductivity observed, is certainly very noteworthy. Through density functional theory calculations and modeling, a distinct topological character is ascertained, which leads to such high AHC. This is proposed as a crucial factor in finding materials with a large anomalous Hall angle.

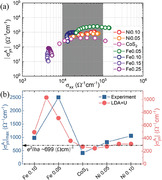

## 1 Introduction

The anomalous Hall effect (AHE) has attracted substantial attention due to its fascinating mechanisms and potential applications in spintronic and memory devices.^[^ ^1^, ^2^ ^]^ The origin of AHE, a subject of extensive studies from a fundamental perspective, is predominantly attributed to Berry curvature (BC).^[^ ^3^, ^4^, ^5^, ^6^ ^]^ The presence of monopole charges at nontrivial band crossings, as key sources of BC, renders topological materials, including Weyl and Dirac semimetals, as ideal systems for observing large AHE.^[^ ^7^, ^8^ ^]^ AHE has been observed in various Weyl systems, starting with identification of the momentum‐space magnetic monopoles in SrRuO_3_.^[^ ^9^ ^]^ Subsequently, exceptionally high AHEs have been detected in magnetic Weyl semimetals such as Co_3_Sn_2_S_2_^[^ ^10^ ^]^ and Co_2_MnAl,^[^ ^11^ ^]^ and a magnetic Weyl nodal line semimetal, Co_2_MnGa.^[^ ^12^ ^]^ Although the critical role of BC sources in intrinsic AHE is acknowledged, an effective strategy to substantially enhance anomalous Hall conductivity (AHC) is still missing. Evidence from angle‐resolved photoemission spectroscopy (ARPES) experiments and density functional theory (DFT) calculations has revealed a wider presence of materials with BC sources than previously anticipated.^[^ ^13^, ^14^ ^]^ However, only a limited number of materials have been identified to exhibit topology‐driven electrical properties, resulting in an estimated AHC of ≈ *e* ^2^/(*ha*), where *a* denotes the out‐of‐plane lattice constant. The small Hall conductance may arise from the dominance of trivial energy states^[^ ^15^ ^]^ or from BC sources located far from the Fermi energy.^[^ ^15^, ^16^ ^]^ Another crucial factor is that BC sources within the momentum space may nullify each other.^[^ ^17^ ^]^ As evidenced in the ferromagnetic body‐centered‐cubic Fe, such counteractive BC contributions to the AHE typically result in a lower‐than‐expected AHC value.^[^ ^18^ ^]^ This trend is common in most materials, often resulting in an AHC that is less than *e* ^2^/(*ha*).

Half‐metals, characterized by a single occupied spin band, significantly reduce the cancellation likelihood, thereby creating a conducive environment for enhanced AHE. Notably, cobalt disulfide (CoS_2_) is of particular interest in this context due to its inherent itinerant ferromagnetism and its potential to exhibit half‐metallicity, as extensively explored in previous studies.^[^ ^19^, ^20^, ^21^, ^22^ ^]^ DFT analyses have demonstrated a minimal overlap between spin majority and minority bands in CoS_2_ due to large exchange splitting,^[^ ^21^ ^]^ and a slight hole doping could induce a half‐metallic state.^[^ ^19^, ^23^, ^24^, ^25^ ^]^ By investigating the effects of Fe doping on various magnetic and electric properties of CoS_2_, complemented by Point Contact Andreev Reflection (PCAR) analysis, they revealed that Fe doping levels above 7% induce a highly spin‐polarized state. Recent findings have further enriched our understanding of CoS_2_, particularly the discovery of magnetic Weyl points near the Fermi level.^[^ ^26^ ^]^ Another study presented correlations of a large linear positive magnetoresistance of CoS_2_ with Berry curvature, suggesting that the masses of Weyl nodes near the Fermi level are key sources of Berry curvature.^[^ ^27^ ^]^

In this research, we focused on Fe content‐dependent transport studies on Co_1 − *x*_Fe_*x*_S_2_ and achieved an exceptionally high intrinsic AHC. Doping is an effective method for adjusting the Fermi level in Co_1 − *x*_(Fe,Ni)_*x*_S_2_ alloys. To examine the impact of chemical doping, the values of *x* and *y* in Co_1 − *x*_Fe_*x*_S_2_ (denoted “Fe*x*”) and Co_1 − *y*_Ni_*y*_S_2_ (denoted “Ni*y*”), respectively, were adjusted within a range where both *x* and *y* ⩽ 0.10. Our DFT and ARPES studies reveal that the dominant contribution to the AHC originates from the BC sources generated by the gapped Dirac dispersions around the *k*_*z*_ = 0 plane, as opposed to the well‐known Weyl nodes. The maximum AHC achieved by modulating the Fermi level through doping, particularly within the Dirac gaps, as validated by theoretical calculations and experimental results. We determined that the interaction between inversion symmetry and *d*‐wave‐like spin‐orbit coupling of spin‐polarized *e*_*g*_‐orbitals results in equal‐sign BC sources over the four quadrants of the Brillouin zone (BZ). Our study may provide a novel materials design strategy to attain a large AHC.

## 2 Results and Discussion

### 2.1 Transport Properties

**Figure 1:**
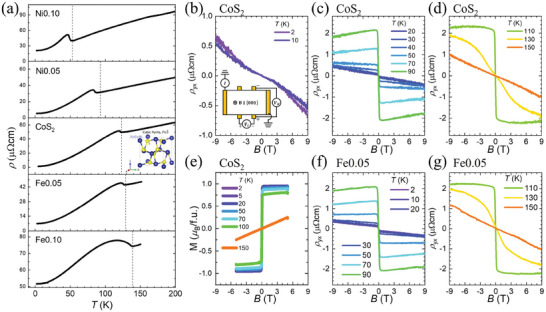
Detailed characterization of pristine and doped CoS_2_. a) Temperature‐dependent longitudinal resistivity, ρ_*xx*_, for pristine CoS_2_ and CoS_2_ doped with Ni and Fe at concentration of 5%, 10% (labeled as Ni0.10, Ni0.05, CoS_2_, Fe0.05, and Fe0.10 for simplicity). Vertical dashed lines indicate the corresponding Curie temperatures *T*_c_ of 53, 93, 123, 129, and 139K, respectively, as determined from SQUID measurements. b–d) Hall resistivity, ρ_*yx*_, of CoS_2_ over three temperature regimes: b) *T* < 20 K, c) 20 K ⩽ *T* < 110K, and d) 110K < *T*. e) Magnetization of CoS_2_ measured up to *B* = 5T at various temperatures, showing saturation at a low magnetic field of 0.3T below *T*_c_. f,g) Hall resistivity of the Fe0.05 in two temperature ranges: f) *T* < 110K and g) 110K ⩽ *T*, with clear saturation observed at all temperatures below *T*_c_. The inset in (b) schematically shows the Hall measurement configuration.

**Figure** **1**a shows temperature‐dependent resistivity ρ_*xx*_ for Ni0.10, Ni0.05, CoS_2_, Fe0.05, and Fe0.10. CoS_2_ has a cubic pyrite crystal structure, which is shown in the inset of Figure 1a.^[^ ^24^, ^28^ ^]^ Vertical dashed lines represent the Curie temperature (*T*_c_) for each sample, specifically at 53, 93, 123, 129, and 139 K, respectively. All samples exhibit metallic behavior across the entire temperature range. Notably, a resistivity anomaly is observed just below *T*_c_, attributable to electron‐magnon scattering.^[^ ^23^, ^25^ ^]^ Fe doping leads to a moderate increase in *T*_*c*_, while Ni doping significantly suppresses it. Both Fe and Ni doping increase the residual resistivity.

The Hall measurement configuration, as depicted in the inset of Figure 1b, enables simultaneous measurements of magnetoresistance and Hall resistivity. The Hall resistivity of CoS_2_ are separately displayed in Figure 1b–d, categorized by temperature regions. In the low temperature region (*T* < 20 K, Figure 1b), the Hall resistivity does not exhibit a clear saturating behavior. In contrast, for the temperature ranges displayed in Figure 1c,d (*T* ⩾ 20 K and *T* = 110K, respectively), saturation in Hall resistivity is observable. In order to separate ordinary Hall effect and anomalous Hall effect, we look for Hall resistivity saturation at the same field as the magnetization saturation. The field‐dependent magnetization *M*(*B*) of CoS_2_, shown in Figure 1e almost entirely saturates near 0.3 T, reaching 0.95 μ_*B*_/Co at *T* = 2 K. This suggests that CoS_2_ is a soft magnet with a small coercive field of approximately 0.005 T. Therefore, the analysis of the anomalous Hall effect in CoS_2_ is restricted to temperatures above 20 K (as shown in Figure 1c,d). For the Fe0.05, as shown in Figure 1f,g, the AHE is distinguishable at all measured temperatures, presenting no issues in analysis. Anomalous Hall resistivity $\rho_{yx}^{A}$ can be derived from $\rho_{yx}=\rho_{yx}^{O}+\rho_{yx}^{A}=R_{o}B+\mu_{0}R_{s}M$ by subtracting ordinary Hall resistivity $\rho_{yx}^{O}$ proportional to *B*. Here, *R*_*o*_ and *R*_*s*_ are ordinary and spontaneous Hall coefficients, respectively.

Although the saturation value of magnetization does not change a lot when the temperature goes up to *T* = 100K, the saturation value of ρ_*yx*_, that is, $\rho_{yx}^{A}$ changes a lot with temperature increasing in Figure 1c. This can be potentially attributed to the increasing *R*_*s*_(*T*) as depicted by Karplus‐Luttinger equation,^[^ ^29^ ^]^

(1) $$R_{s}\left(T\right)=S_{H}\rho_{xx}^{2}\left(T\right)$$

where *S*_*H*_ denotes the Hall factor.^[^ ^30^ ^]^ Notably, the Karplus‐Luttinger equation overlooks the lattice disorder and it predicts only intrinsic AHE.^[^ ^3^, ^10^ ^]^ Consequently, $\rho_{yx}^{A}$ attains a maximum at 100K, suggesting an intrinsic band origin of the AHE below *T*_c_. This behavior should result in a plateau‐like AHC within the limit of ρ_*yx*_ ≪ ρ_*xx*_, as observed in several itinerant ferromagnets.^[^ ^10^, ^31^, ^32^, ^33^, ^34^ ^]^ In such cases, the following approximation can be used:^[^ ^32^ ^]^

(2) $$\sigma_{yx}^{A}≅-\rho_{yx}/\rho_{xx}^{2}≅-\mu_{0}S_{H}M$$

**Figure 2:**
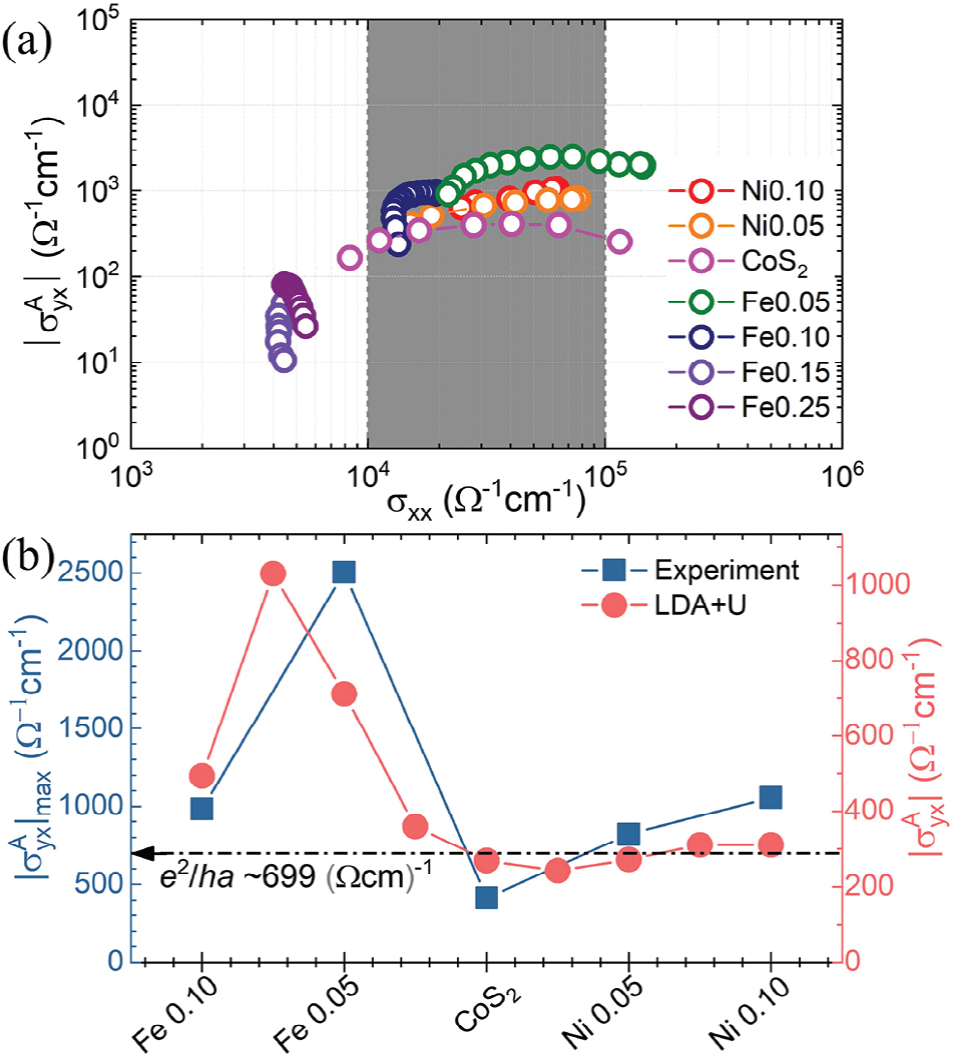
a) A log‐log scale plot of $|\sigma_{yx}^{A}|$ versus σ_*xx*_. The intermediate regime of σ_*xx*_ is marked in dark gray as the intrinsic AHE region. b) The maximum value of $|\sigma_{yx}^{A}|$ obtained from (a) for various Fe or Ni doping (blue squares). The dash‐dot line refers to the expectation value of AHC when the Fermi level is located at a single BC source gap of which the anticrossing of linear band dispersion gapped by spin‐orbit coupling, using a lattice constant of CoS_2_, *a* = 5.54Å. By plotting double‐Y graph, $|\sigma_{yx}^{A}|$ calculated from the band structure of CoS_2_ is shown as red circles. $|\sigma_{yx}^{A}|$ is obtained by changing upper limit of integration, which is the same as Fermi level changing.

**Figure** **2**a shows $|\sigma_{yx}^{A}|$ vs σ_*xx*_ of Fe‐ and Ni‐doped CoS_2_ in a log–log scale. A plateau region is apparent for CoS_2_ and also for low‐doped ferromagnets (up to Fe0.10 and Ni0.10). The plateau‐like behavior in the region marked by the dark gray background color strongly suggests the intrinsic nature of the observed AHE (originating from the BC). Highly Fe‐ or Ni‐doped CoS_2_ does not exhibit such plateau‐like behavior and is not discussed in this study. In the Supporting Information (see Section S8, Supporting Information), we have advanced our methodology beyond the basic log‐scale plot. This refined analysis reveals that the extrinsic contribution is primarily relevant at high temperatures. In contrast, the anomalous Hall conductivity (AHC) at low temperatures is predominantly intrinsic, thereby having a negligible impact on the overall findings of our study.

Figure 2b illustrates $|\sigma_{yx}^{A}{|}_{\max}$, the maximum value of $|\sigma_{yx}^{A}|$ which is the intrinsically dominating AHC,^[^ ^30^ ^]^ for various Fe or Ni doping values in the itinerant ferromagnetic regime. We first note that the AHC data peaks at Fe0.05. In topological metals, such peaky behavior can be realized with a band‐crossing point where density of states (DOS) vanishes.^[^ ^4^ ^]^ If we assume a single BC source in our system, the plateau‐like AHC should display a saturation value of $|\sigma_{yx}^{A}|≅e^{2}/\left(ha\right)\approx 699\;\Omega^{-1}{\mathrm{cm}}^{-1}$ (dash‐dot line in Figure 2b), where the CoS_2_ lattice constant *a* is taken as 5.54Å.^[^ ^35^ ^]^ However, Fe0.05 exhibits a peak value of 2507 Ω^−1^cm^−1^, more than four times larger than that of CoS_2_. Generally, an AHC ranges between 100 and 1000 Ω^−1^cm^−1^,^[^ ^10^, ^32^ ^]^ with the highest reported AHC value of approximately 2000 Ω^−1^cm^−1^ in Fe crystals.^[^ ^32^ ^]^ Notably, the achieved $|\sigma_{yx}^{A}|$ value of 2507 Ω^−1^cm^−1^ in Fe0.05 sets a new record. Furthermore, the LDA+*U* calculated $|\sigma_{yx}^{A}|$ value closely mirrors our experimental data, strongly suggesting a rigid shift in the band structure achieved through chemical doping.

### 2.2 First‐Principles Calculations

**Figure 3:**
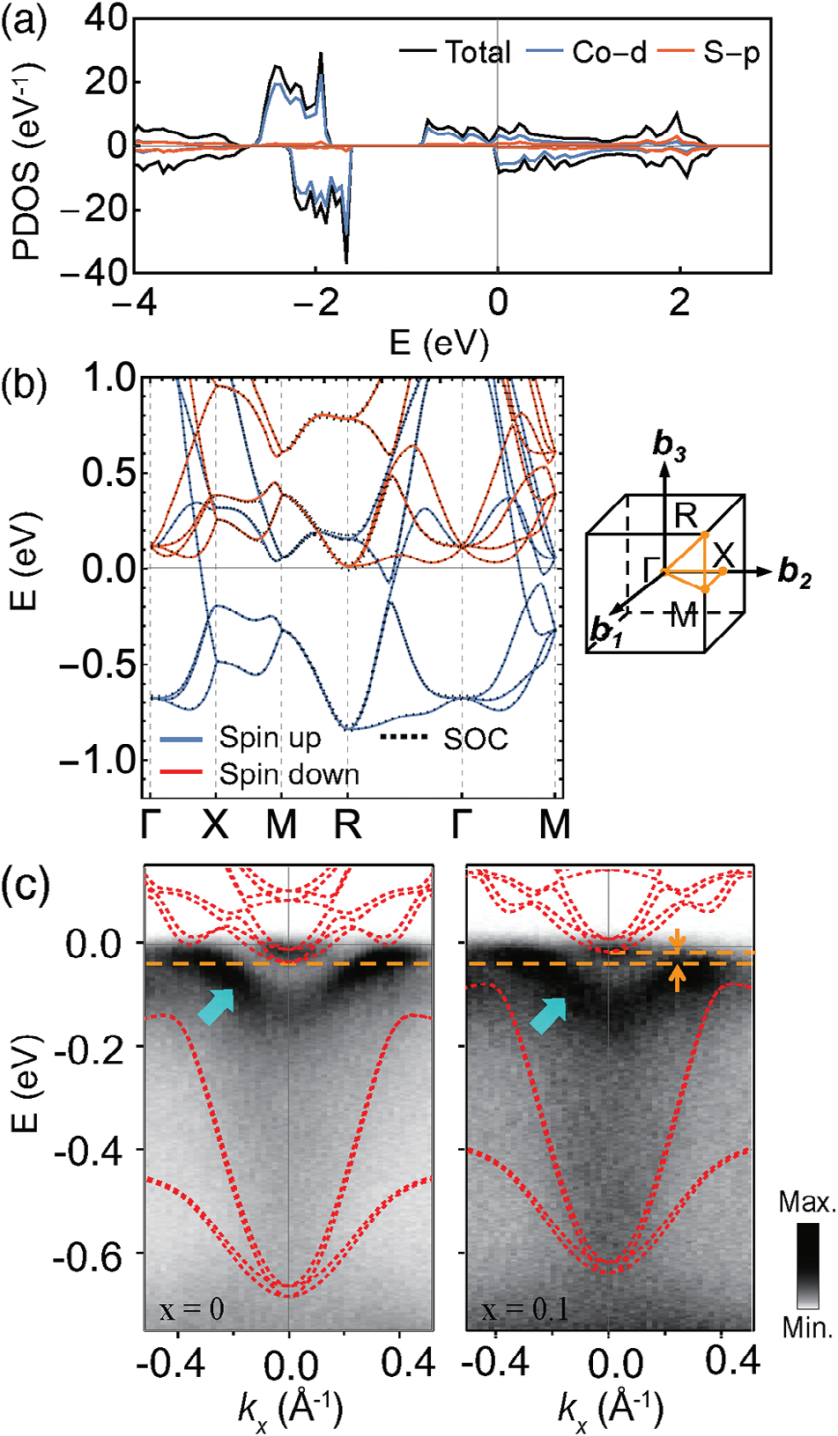
a) Orbital‐projected partial density of state. b) Band structures along high‐symmetry lines. c) ARPES data compared with the DFT band structures (red dashed lines) along the *Z* − *R* − *Z* line of Co_1 − *x*_Fe_*x*_S_2_. Left: *x* = 0 aligned with the DFT band of pristine CoS_2_; right: *x* = 0.1 aligned with 0.1h/formula unit doped CoS_2_. For a clear comparison, band energy was renormalized with a factor of 1.3 and the Fermi energy was shifted up by 40 meV for both CoS_2_ and Fe0.10 cases. The bands denoted with cyan arrows are surface bands. The orange arrows denote the energy shift of DFT bands for the electron pocket at the *R*‐point.

A significant variation in the AHC concerning electron or hole doping was observed, displaying a fourfold difference between the largest and smallest values. Given that $\sigma_{yx}^{A}$ is insensitive to σ_*xx*_, the doping‐induced change in the electronic structures was the primary cause for the differences in AHC. **Figure** **3** presents the electronic structures calculated using the LDA+*U* scheme. The partial density of states (PDOS) shows the nearly half‐metallic nature of the system, with the frontier orbitals mainly derived from Co $d_{x^{2}-y^{2}}$ and $d_{z^{2}}$ orbitals from the Co coordination, approximately considered as a CoS_6_ octahedron,^[^ ^26^ ^]^ with *t*_2*g*_‐*e*_*g*_ splitting. The band structures drawn along the high‐symmetry lines reveal that the spin‐orbit coupling has no significant effect because the centers of the Dirac dispersions, most sensitive to the spin‐orbit coupling, are shifted away from the high‐symmetry lines due to the mirror symmetry breaking. Importantly, a gapped band crossing at approximately −60 meV near the *M*‐point with the gap opening from the mirror symmetry breaking was observed along the Γ‐*M* high symmetry line, which will be discussed in detail later.

A comparison between the ARPES and DFT band structures indicates that the primary change in the band structure due to doping arises from the shift in the chemical potential. Figure 3c presents a comparison of the ARPES band structures of CoS_2_ and Fe0.10 with those calculated with corresponding chemical potential shifts. The ARPES band structures around the *R*‐points display three primary features: a small minority bulk electron pocket at the *R*‐point, a surface band around −0.1 eV denoted as cyan arrows, and large dispersive bulk bands within the energy range of approximately −0.1 and −0.7 eV. We note that there are surface states present (marked with cyan arrow) in addition to the bulk states that are discussed in a previous study.^[^ ^26^ ^]^ The DFT band align well with the ARPES data with a mass renormalization of m* = 1.3 *m*_DFT_. Importantly, a chemical potential shift of approximately 30 meV (indicated by the orange arrows in Figure 3c) was observed in both the Fe‐doped ARPES and hole‐doped DFT bands, suggesting that the primary effect of doping can be considered a shift in the chemical potential. The alterations in the band structures due to the chemical potential shift are further corroborated by comparing the calculated and experimental AHC values in Figure 2b. The measured AHC values for both Ni‐ and Fe‐doped CoS_2_ align well with those obtained from the DFT calculations. We note that the AHC values calculated using the virtual crystal approximation (VCA) also show similar doping dependence in which the effect of doping is considered by mixing the Co pseudopotential with Ni and Fe ones. This is further supported by the fact that the effect of the pseudopotential mixing on the band structures can be mainly considered as the chemical potential shifts near the Fermi energy. (see Figure S4, Supporting Information for details.)

**Figure 4:**
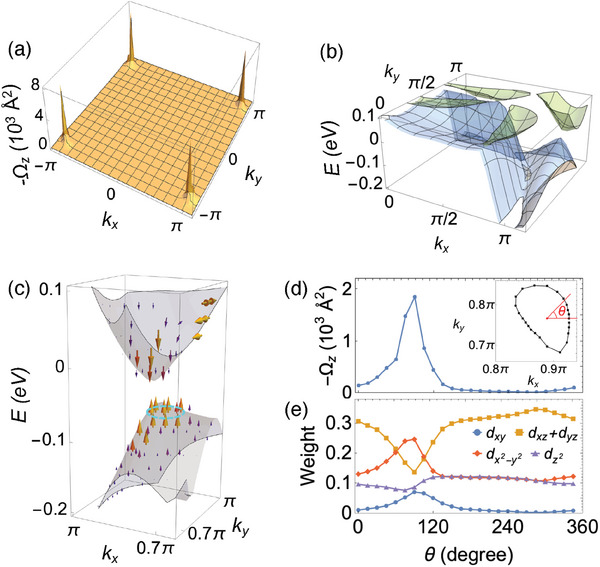
a) Momentum‐resolved out‐of‐plane BC integrated up to ‐60 meV below the Fermi energy. b) Band structures evaluated in the *k*_*z*_ = 0 plane. c) BC of the bands near the *M*(π, π, 0)‐point. The gray surfaces denote *E*(**k**) in the *k*_*z*_ = 0 plane, and BCs are represented with arrows. d) *z*‐component of the BC of the states along the cyan‐colored ring from (c). The states are parameterized as θ defined, showing in the inset a top view of the ring and its center. e) Orbital weight of the states along the ring.

Further investigation of momentum‐dependent BCs reveals four distinct BC hotspots in the BZ, all exhibiting the same sign, which primarily contributes to the large AHC. For pristine CoS_2_, the AHC evaluated by varying the Fermi energy displays a peak value at ‐60 meV (See Figure S5, Supporting Information for detail), in which the AHC as a function of the energy is consistent with the previous report.^[^ ^27^ ^]^ The momentum‐dependent out‐of‐plane BC (Ω_*z*_) in the *k*_*z*_ = 0 plane integrated up to ‐60 meV was then calculated, as shown in **Figure** **4**a. Four hotspots were observed near *M*‐points; therefore, the BC is mainly contributed from the region around *M*(π, π, 0)‐points with small *k*_*z*_ values, given the strong suppression of BC around *k*_*z*_ = π plane and much smaller peak values in the BC around the Weyl points observed in the *M*(0, π, π)‐points in the *k*_*x*_ = 0 plane (see Sections S2 and S3, Supporting Information for details). Unlike typical ferromagnetic metals such as Fe, a remarkable feature of a momentum‐dependent BC is that, all hotspots peaking in the BC share the same sign, and the lack of cancellation results in a substantial net AHC. The band structures in the *k*_*z*_ = 0 plane (Figure 4b) demonstrate that the BC peak is linked with the gapped band crossing along the Γ‐*M* line. Analyses of the BC of states around the gapped band crossing Figure 4c indicate that the BCs, primarily along the out‐of‐plane direction, exhibit opposite directions between the lower and upper energy bands. The AHC is expected to be highest when the chemical potential is positioned inside the gap. This was confirmed by the band structures with 7.5% hole doping, where the Fermi energy is located between the gap (see Section S1, Supporting Information for details).

Figure 4d,e display the BC and Co‐*d* orbital characters of the states around the gapped band crossing, showing the correlation between the BC and orbital characters. Large variations in both the BC and orbital characters of the states along the ring in the lower band (the cyan ring in panel (c)) parameterized with respect to the angle θ (inset in panel (d)) are observed. Notably, a peak in the BC at approximately θ = 100° is detected.

The calculated *e*_*g*_ orbital characters for the corresponding states also display a peak in the $d_{x^{2}-y^{2}}$ and *d*_*xy*_ orbitals at around the same θ. Combined with the relatively θ‐insensitive $d_{z^{2}}$ orbital weight and a dip in the *d*_*xz*_/*d*_*yz*_ orbital weight at around θ = 100°, our analysis suggests that orbital mixing between the $d_{x^{2}-y^{2}}$ and *d*_*xy*_ orbitals is key to understanding the large BC around the gapped‐band crossing. Based on the DFT calculation results, the mechanism generating a large BC with the same sign throughout the BZ was explored using a tight‐binding analysis of the frontier orbitals.

### 2.3 Tight‐Binding Analysis

**Figure 5:**
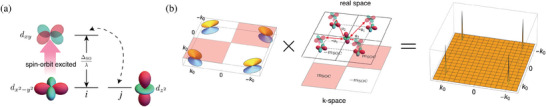
a) Effective hopping between $d_{z^{2}}$ and $d_{x^{2}-y^{2}}$ orbitals mediated by the SOC. b) Illustration of the method used to obtain a uniform BC structure whose peaks have the same sign over the BZ. In the left and middle panels, the colors on the momentum space quadrants represent different signs of $\Gamma_{0}^{\left(j\right)}$ and SOC‐induced mass $m_{\mathrm{SOC}}^{\left(j\right)}$. The BC, which is a product of $\Gamma_{0}^{\left(j\right)}$ and $m_{\mathrm{SOC}}^{\left(j\right)}$, exhibits four peaks with the same sign.

We demonstrate that if four Dirac nodes in four momentum‐space quadrants are gapped by a *d*_*xy*_‐wave‐like mass term (proportional to (−1)^*j*^, with the quadrant number *j*), the BCs around these four Dirac nodes share the same signs, as revealed through a tight‐binding analysis. As a result, the AHC can be finite without cancellation of the BCs in different quadrants. To understand this, we start from a 2 × 2 effective Dirac Hamiltonians in four quadrants (labeled by *j*) of the BZ. This is given by

(3) $$\begin{array}{c} H_{0,\mathrm{eff}}^{\left(j\right)}=\sum_{\alpha =x,z}f_{0,\alpha }^{\left(j\right)}\left(q\right)\sigma_{\alpha } \end{array}$$

with Pauli matrices σ_α_ and real‐valued functions $f_{0,\alpha }^{\left(j\right)}$. A finite BC can be obtained through the gap‐opening achieved by adding a mass term $m_{\mathrm{SOC}}^{\left(j\right)}\sigma_{y}$ introduced by the spin‐orbit coupling (SOC). Here, $m_{\mathrm{SOC}}^{\left(j\right)}\propto {\left(-1\right)}^{j}$ because the SOC‐induced hybridization between neighboring $d_{x^{2}-y^{2}}$ and $d_{z^{2}}$ orbitals is mediated by *d*_*xy*_‐orbital at higher energy (**Figure** **5**a). Then, the BC in the *j* ^th^ quadrant is given by

(4) $$\begin{array}{c} \Omega_{z}^{\left(j\right)}\left(q\right)\approx \frac{m_{\mathrm{SOC}}^{\left(j\right)}}{|m_{\mathrm{SOC}}^{\left(j\right)}{|}^{3}}\Gamma_{0}^{\left(j\right)}\left(q\right) \end{array}$$

where $\Gamma_{0}^{\left(j\right)}\left(q\right)=\partial_{q_{y}}f_{0,z}^{\left(j\right)}\partial_{q_{x}}f_{0,x}^{\left(j\right)}-\partial_{q_{x}}f_{0,z}^{\left(j\right)}\partial_{q_{y}}f_{0,x}^{\left(j\right)}$. (See Section S6, Supporting Information for details.) Since $H_{0,\mathrm{eff}}^{\left(j\right)}$ respects mirror symmetry, we have $\Gamma_{0}^{\left(j\right)}\left(q\right)\propto {\left(-1\right)}^{j}$, as illustrated in Figure 5b. As a result, BC($\propto m_{\mathrm{SOC}}^{\left(j\right)}\Gamma_{0}^{\left(j\right)}\left(q\right)$) has the same sign for all quadrants; the Hall conductivity, an integral of BC over the BZ, can be nonzero. Given that a spin‐polarized system was considered regarding the half‐metallicity of CoS_2_, the cancellation of BCs between the two spin species can be ruled out.

## 3 Conclusion

We investigated the AHC of CoS_2_ by tuning its chemical potential through hole (Fe) and electron (Ni) doping. The plateau‐like AHC observed for doping levels of up to 10% in both Fe and Ni cases signifies an intrinsic AHE, stemming from the BC‐induced deflection of charge carriers. The AHC, peaking at Fe0.05, recorded the highest value among the intrinsic origins. Although it is well known that CoS_2_ hosts eight Weyl points near the *k*_*z*_ = π plane, acting as the BC sources, these points cannot be considered as the origin of the observed colossal AHC due to the alternating signs of their monopole charges. Instead, we propose that the AHC originates from four other BC hotspots situated around the *k*_*z*_ = 0 plane, as determined from DFT calculations. These four points are represented by massive Dirac cones centered near **k** = (± π, ±π, 0).

The contribution to the AHC from these new hotspots can become large at specific doping levels because the corresponding BC exhibits the same sign around these four Dirac nodes. We demonstrated that this feature of BC can emerge when the inversion‐symmetric and spin‐polarized Dirac dispersions are gapped by a *d*‐wave‐like SOC, which arises when the SOC between *e*_*g*_ orbitals at neighboring sites is mediated by *d*_*xy*_‐orbitals.

Our study unveils the underlying mechanism of the huge AHC in CoS_2_ and illuminates an effective strategy for searching for materials with similar properties by tuning the doping level and designing materials with the desired orbital characteristics.

## 4 Experimental Section

### Single Crystal Growth

Co_1 − *x*_Fe_*x*_S_2_ (*x* = 0, 0.05, 0.10, 0.15, and 0.25), and Co_1 − *y*_Ni_*y*_S_2_ (*y* = 0.05, and 0.10) were successfully grown using the chemical vapor transport (CVT) method, employing TeCl_4_, as the transporting agent. The process involved sealing a mixture of polycrystalline powders and TeCl_4_ in an air‐evacuated quartz tube. This tube was then placed in a two‐zone furnace. The center temperature of the quartz tube was maintained at 750–825 °C with a temperature gradient of 2 °C cm^−1^ for four weeks. Crystals with typical sizes of 0.5–2 mm^3^ were obtained.

### Transport Measurements

Both longitudinal (*V*_*l*_) and Hall (*V*_*H*_) voltage drops were measured simultaneously in the Hall measurement configuration, as demonstrated in the inset of Figure 1a. CoS_2_ single crystals were carefully cut along the (001) plane to a thickness of tens of micrometers using a razor blade. These crystals typically measured about 1 × 0.5 × 0.04mm^3^. Six Au wires were affixed to the crystal using Ag paste (Dupont 5065). A 5mA DC source from Keithley 224 was used, and voltages in both longitudinal and transverse directions were recorded with a Keithley 34420A nanovoltmeter. To counteract electrothermal effects, voltages were measured multiple times with reverse currents and then averaged.

The longitudinal resistivity (ρ_*xx*_) and Hall resistivity (ρ_*yx*_) were obtained through symmetrization and anti‐symmetrization, respectively. The magneto‐ and Hall resistance were measured by increasing and decreasing magnetic fields at rates of 0.5 Tmin^−1^. To compensate for the misalignment of the electrodes, magneto‐(Hall) resistance was symmetrized(anti‐symmetrized) from longitudinal(transverse) voltage as follows:

(5) $$\begin{array}{c} R_{xx1}=\left(R_{xx,+}\left(B\right)+R_{xx,-}\left(-B\right)\right)/2 \end{array}$$

(6) $$\begin{array}{c} R_{xx2}=\left(R_{xx,+}\left(-B\right)+R_{xx,-}\left(B\right)\right)/2 \end{array}$$

(7) $$\begin{array}{c} R_{yx1}=\left(R_{yx,+}\left(B\right)-R_{yx,-}\left(-B\right)\right)/2 \end{array}$$

(8) $$\begin{array}{c} R_{yx2}=\left(R_{yx,-}\left(-B\right)-R_{yx,+}\left(B\right)\right)/2 \end{array}$$

The +(−) sign in the subscript represents the increasing(decreasing) applied magnetic fields. A ±9T magnetic field was applied using a NbTi superconducting magnet. The temperature of the sample was controlled using the various temperature input(VTI) systems of the cryogen‐free measurement system(CFMS, Cryogenics Ltd).

### Magnetic Property Measurements

Molar susceptibility was measured along the *a*‐axis under a magnetic field of 0.1T using a superconducting quantum interference device magnetometer (MPMS, Quantum Design).

### Angle‐Resolved Photoemission Spectroscopy (ARPES) Measurements

ARPES measurements were conducted at the beamlines 4.0.3 and 7.0.1 of the Advanced Light Source, Lawrence Berkeley National Laboratory. Samples were cleaved *insitu* and measured at 20 K in an ultra‐high vacuum better than 5 × 10^−11^ torr.

### First‐Principles Calculations

First‐principle DFT calculations were performed within the local density approximation plus *U* (LDA+*U*) scheme using the Vienna ab initio simulation package (VASP).^[^ ^36^, ^37^ ^]^ The projector augmented wave (PAW) method,^[^ ^38^ ^]^ and the Ceperley‐Alder exchange‐correlation functional^[^ ^39^ ^]^ were employed, with an energy cutoff of 500 eV and *k*‐point sampling of 8 × 8 × 8 for a 12‐atom unit cell. Semicore *s*‐ and *p*‐ orbitals were included for Co pseudopotential. The experimental atomic structure was used.^[^ ^20^ ^]^ The rotationally invariant form of on‐site Coulomb interaction was used,^[^ ^40^ ^]^ parameterized by *U* = 2.25eV and *J* = 0.5eV for Co‐*d* orbitals. This approach reproduced the nearly half‐metallic ground state with a magnetization value of 0.92 μ_*B*_/Co, similar to the experimental data of approximately 0.9 μ_*B*_/Co,^[^ ^23^, ^41^, ^42^ ^]^ as well as our low‐temperature magnetization measurements (0.95 μ_*B*_/Co). The charge‐only LDA exchange‐correlation functional was used with the plus *U* extension, which delivered the correct trend of the exchange splitting proportional to *J* for ferromagnetic metals.^[^ ^43^, ^44^ ^]^ Spin‐orbit coupling was included. For the calculations of Fe‐ and Ni‐doped CoS_2_, the corresponding hole or electron doping was implemented by adjusting the total number of electrons with compensating uniform background charge. In addition, the band structures and AHC were calculated using the VCA implemented in VASP^[^ ^45^, ^46^ ^]^ to include the doping effect and were compared with those obtained by chemical potential shits. In the VCA calculations, semicore *s* and *p* states were not considered in Fe, Co, and Ni pseudopotentials. The AHC was computed using Wannier90^[^ ^47^ ^]^ and the Wannier‐Berri code^[^ ^48^, ^49^ ^]^ in which the tight‐binding Hamiltonian was constructed using Co‐*d* and S‐*p* derived bands.

## Conflict of Interest

The authors declare no conflict of interest.

## Supporting information

Supporting Information

## Data Availability

The data that support the findings of this study are available from the corresponding author upon reasonable request.
