# Supplementary material for: Tunable Colossal Anomalous Hall Conductivity in Half‐Metallic Material Induced by d‐Wave‐Like Spin‐Orbit Gap
Source: Adv Sci (Weinh). 2024 Mar 21;11(20):2307288. doi: 10.1002/advs.202307288 (PMC11132085; doi:10.1002/advs.202307288)
Supplement: Supplementary file 1 — Supporting Information [file ADVS-11-2307288-s001.pdf]

ADVANCED SCIENCE
Open Access

Supporting Information

for *Adv. Sci.*, DOI 10.1002/advs.202307288

Tunable Colossal Anomalous Hall Conductivity in Half-Metallic Material Induced by *d*-Wave-Like Spin-Orbit Gap

Joonyoung Choi, Jin-Hong Park, Wonshik Kyung, Younsik Kim, Mi Kyung Kim, Junyoung Kwon, Changyoung Kim, Jun-Won Rhim, Se Young Park* and Younjung Jo**

Supporting Information: Tunable colossal anomalous Hall conductivity in half-metallic material induced by d-wave-like spin-orbit gap

Joonyoung Choi Jin-Hong Park Wonshik Kyung Younsik Kim Mi Kyung Kim Junyoung Kwon Changyoung Kim Jun-Won Rhim Se Young Park* Younjung Jo**

J. Choi, Prof. Y. Jo
Department of Physics, Kyungpook National University, Daegu 41566, Korea
Email Address: jophy@knu.ac.kr
Dr. J.-H. Park, Prof. J.-W. Rhim
Research Center for Novel Epitaxial Quantum Architectures, Department of Physics, Seoul National University, Seoul, 08826, Korea
Email Address: jwrhim@ajou.ac.kr
Dr. W. Kyung, Dr. Y. Kim, Prof. C. Kim
Center for Correlated Electron Systems, Institute for Basic Science, Seoul 08826, Korea
Dr. W. Kyung, Dr. Y. Kim, Prof. C. Kim
Department of Physics and Astronomy, Seoul National University, Seoul 08826, Korea
Dr. M. K. Kim
Department of Physics, Yonsei University, Seoul 03722, Korea
Dr. J. Kwon
Department of Physics, Pohang University of Science and Technology, Pohang 37673, Korea
Prof. J.-W. Rhim
Department of Physics, Ajou University, Suwon 16499, Korea
Prof. S. Y. Park
Department of Physics and Origin of Matter and Evolution of Galaxies (OMEG) Institute, Soongsil University, Seoul 06978, Korea
Prof. S. Y. Park
Integrative Institute of Basic Sciences, Soongsil University, Seoul, 06978, South Korea
Email Address: sp2829@ssu.ac.kr

Keywords: *Anomalous Hall effect, Berry curvature, Half-metals*

1 Doping-dependent band structures

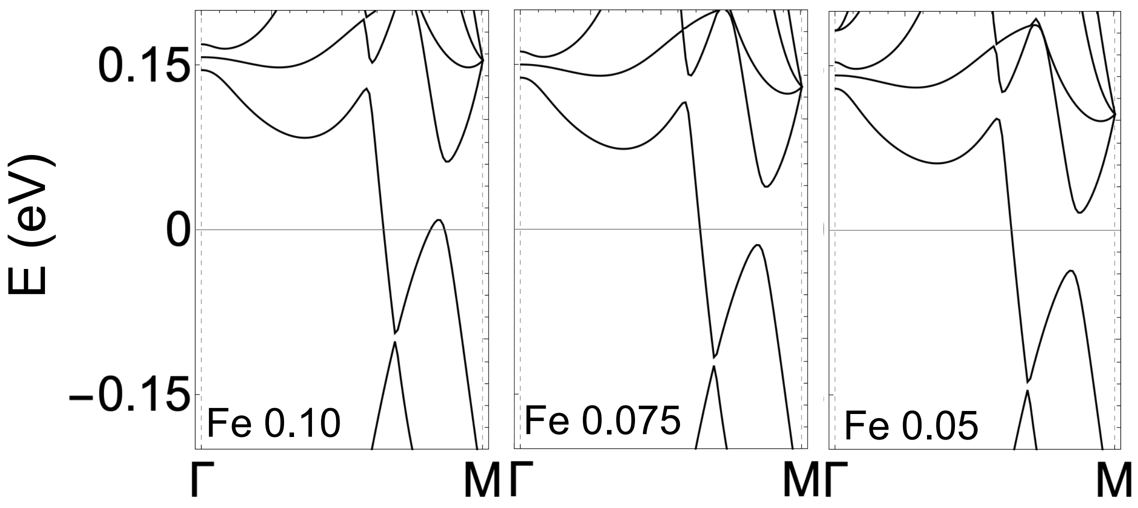

Figure S1. Doping-dependent band structures along the $\Gamma - M$ high-symmetry line.

Fig. S1 illustrates DFT band structures obtained along the $\Gamma - M$ high-symmetry line, showing their variation with respect to doping levels. We observed that at the doping level corresponding to the calculated peak of AHC (i.e., at Fe 0.075), the Fermi energy resides just above the intersection of the gapped band crossing. A decline in the AHC is observed in two distinct scenarios. First, when excluding states from the lower section of the band crossing (evident at Fe 0.10), and second, when incorporating states from the upper portion of the band crossing, which exhibit an opposite sign. This observation is consistent with the momentum-dependent BC, as illustrated in Fig. 4.

2 k_z-dependent band structures and Berry curvature

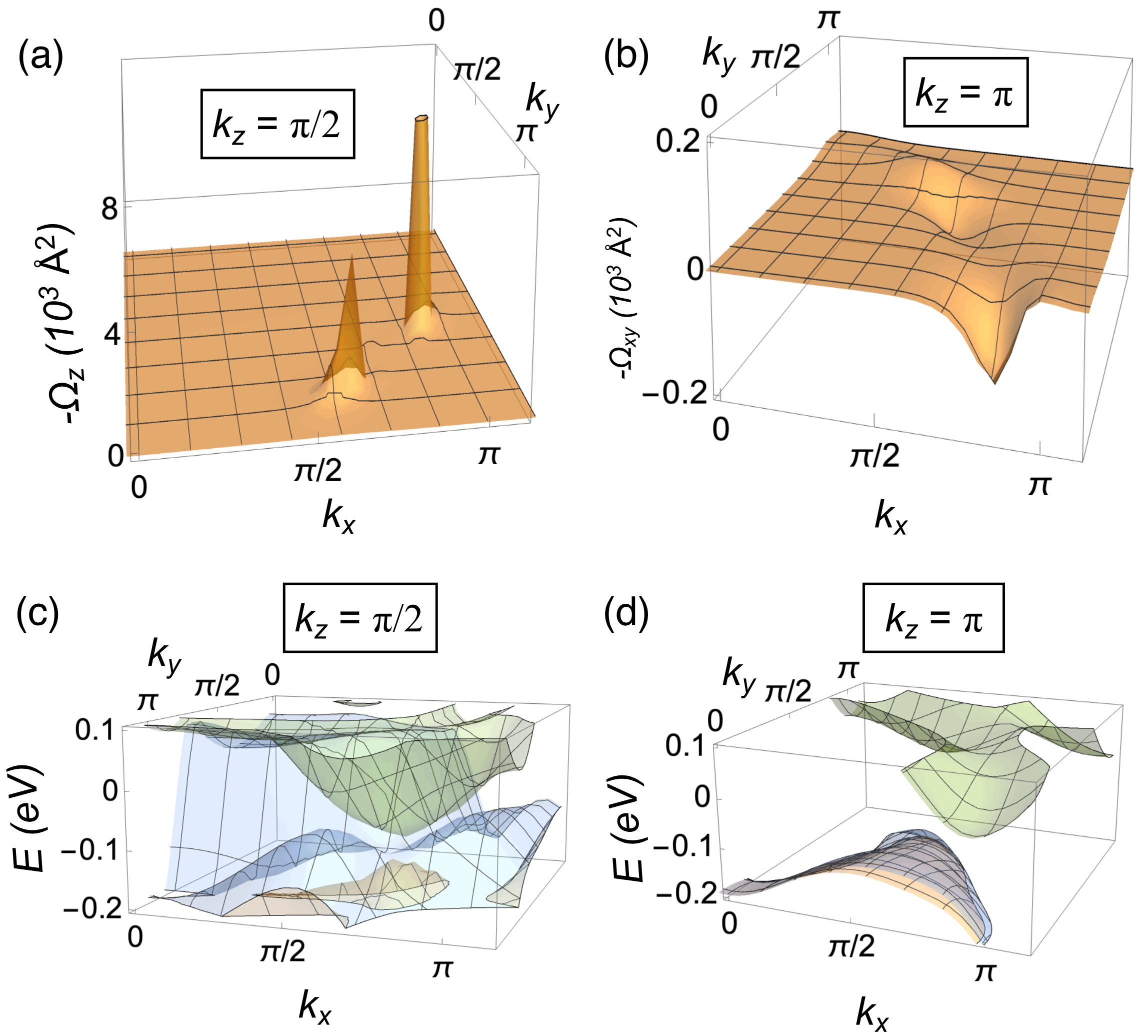

Figure S2. Momentum-resolved out-of-plane BC integrated up to $-60\,\text{meV}$ below the Fermi energy evaluated in the (a) $k_z = \pi/2$ and (b) $k_z = \pi$ planes. Band structures evaluated in the (c) $k_z = \pi/2$ and (d) $k_z = \pi$ planes.

Fig. S2 shows the BC and band structures depending on the out-of-plane crystal momentum k_z. We observed two peaks in the BC around the M-point when $k_z = \pi/2$, where gapped band crossings occur. However, the BC is significantly suppressed for $k_z = \pi$, suggesting that the BC is mainly contributed around the $k_z = 0$ plane.

3 Momentum-resolved Berry curvature in $k_x = 0$ plane

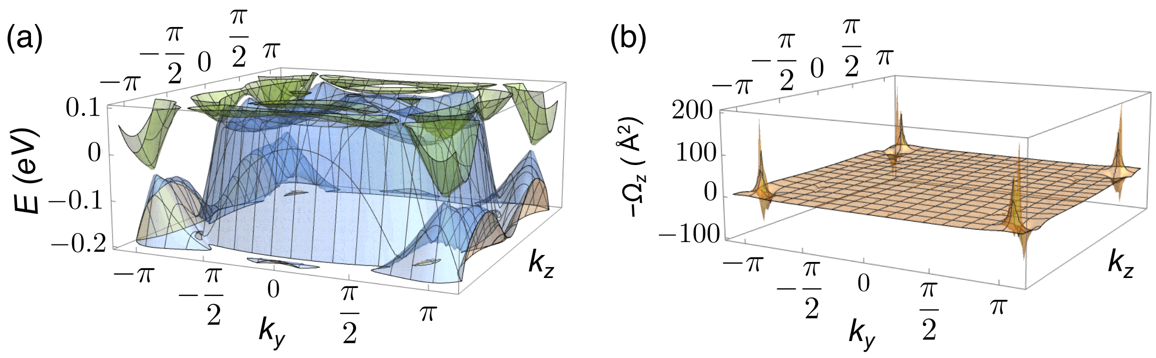

Figure S3. (a) Band structures around Fermi energy in the $k_x = 0$ plane. (b) Momentum-resolved out-of-plane Berry curvature (in Å^2) integrated up to -60 meV below the Fermi energy.

We investigate the band structures and momentum-dependent Berry curvature in the $k_x = 0$ plane, presented in Fig. S3. We find four Weyl points in the $k_x = 0$ plane, consistent with previous reports,[1–3] and also find the peak in the Berry curvature at around the Weyl points. However, the value of the peak is about 40 times smaller than those in the $k_z = 0$ plane ($-\Omega_z$ of 190 Å^2 versus 7887 Å^2), and there is a negative contribution present around the Weyl points with a peak value of -76 Å^2. Although there is a net Berry curvature contributed around the Weyl points that are in line with previous literature, we expect that the major contribution is from the gapped band crossing in the $k_z = 0$ plane.

4 Band structures and doping-dependent AHC using virtual crystal approximation

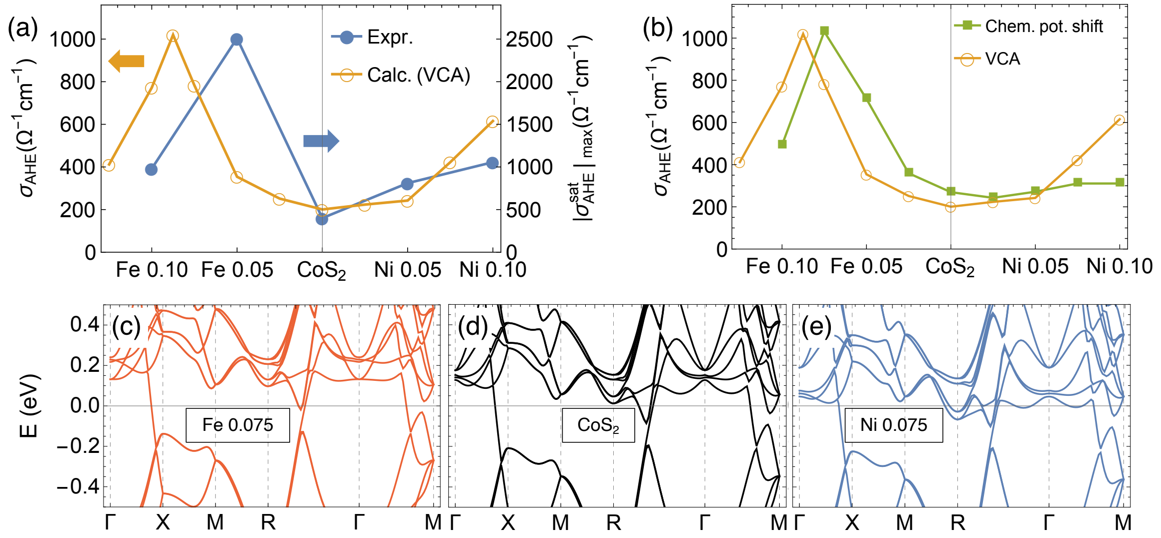

Figure S4. AHC obtained by VCA compared with (a) experimental and (b) theoretical values calculated by chemical potential shift. Band structures calculated by VCA for (c) Fe 0.075, (d) undoped, and (d) Ni 0.075 doped cases. We note that there is a small difference between the CoS_2 band structures in panel (d) and Fig. 2 (b) since in the calculations with VCA, Co pseudopotential without semicore s and p states are used.

In order to check the validity of treating the doping by chemical potential shifts (or equivalently changing the electron number), the electronic structures and AHC using the VCA is calculated, which is a reasonable way to include the effect induced by atomic species change given the similar atomic numbers between Fe, Co, and Ni. We note that the previous DFT calculations of $Co_{1-x}Fe_xO_2$ using the VCA give magnetization values in good agreement with those from experiments and from supercell calculation.[4,5] Fig. S4 (a-b) shows the comparison between experimental and theoretical AHC calculated by chemical potential shifts and by VCA. We find that the overall dependence of AHC on doping is maintained, especially the peak in the AHC for the Fe-doped case. Moreover, the comparison between the band structures obtained by VCA shows that the effect of the doping, within the limit of the VCA, is mainly considered as the rigid band shift, providing support for treating the doping by chemical potential shifts. We note that around the peak AHC value, the Fermi energy is located within the gapped band crossing around the M-point (Fig. S4 (c)), consistent with those calculated by chemical potential shifts.

5 Energy versus anomalous Hall conductivity calculated for CoS_2

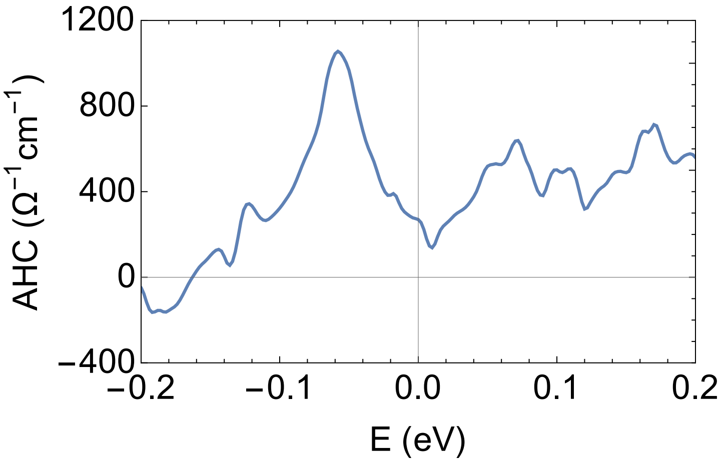

Figure S5. Energy-dependent AHC calculated for CoS_2 obtained by integrating the Berry curvature up to the energy E. The Fermi energy is set to zero.

We calculate the energy-dependent AHC calculated for CoS_2 and find that there is a peak in the AHC around -60 meV, with a peak value of 1056 $\Omega^{-1}\text{cm}^{-1}$. The overall features of AHC with respect to the energy are consistent with the previous report.[1] From the energy dependence, we expect a large increase in AHC with hole doping, consistent with both the experimentally measured and calculated doping-dependent AHC, having a peak value as the amount of hole doping or negative chemical potential shift increases.

6 Tight-binding analysis

In this section, we focus on elucidating the process of deriving BCs, which exhibit equal-signed peaks across the entire BZ. This is achieved by integrating SOC into spin-polarized Dirac dispersions, wherein the nodes are safeguarded by mirror symmetry. In order to encapsulate the fundamental characteristics of CoS_2, we construct a rudimentary conceptual model as outlined below. Initially, we contemplated a two-dimensional (2D) rock-salt lattice structure, as exemplified in Fig. 5(b). This reflects the three-dimensional (3D) rock-salt structural composition of CoS_2. Given that the BC of CoS_2 manifests a peak at the $k_z = 0$ plane, contributing significantly to the AHE, we reduce the dimensions to two in order to describe the effective 2D system positioned at the $k_z = 0$ plane. Subsequently, based on the DFT calculations, we utilize d_{z^2} and $d_{x^2-y^2}$ as the foundation orbitals at each lattice site. The final assumption is that the model Hamiltonian is spin-polarized, reflecting that CoS_2 is half-metallic. We commence with a 4×4 tight-binding model on the 2D rock-salt lattice, excluding SOC, but hosting Dirac dispersions in proximity to the Fermi level. This model comprises two sublattice sites, labeled A and B, where the two d-orbitals are located. The corresponding Bloch Hamiltonian, which includes up to the next nearest hopping processes, assumes the following form:

$$H_0(\mathbf{k}) = \begin{pmatrix} h_{\mathrm{AA}}(\mathbf{k}) & h_{\mathrm{AB}}(\mathbf{k}) \\ h_{\mathrm{BA}}(\mathbf{k}) & h_{\mathrm{BB}}(\mathbf{k}) \end{pmatrix}, \tag{1}$$

where h_{AA}, h_{AB}, and h_{BB} are 2×2 matrices. The submatrices $h_{\mathrm{AA}}(\mathbf{k}) = -\epsilon_0\sigma_0 + \epsilon_d(\mathbf{k})\sigma_z + v(\mathbf{k})\sigma_x$ and $h_{\mathrm{BB}}(\mathbf{k}) = \epsilon_0\sigma_0 + \epsilon_d(\mathbf{k})\sigma_z + v(\mathbf{k})\sigma_x$ describe the hopping processes within the same sublattices, where $\epsilon_d(\mathbf{k}) = t_0(\cos k_x + \cos k_y)$ and $v(\mathbf{k}) = t_1(\cos k_x - \cos k_y)$. We assume that A and B sites generally exhibit different on-site potential $\pm\epsilon_0$. Moreover, for simplicity, we discard the term proportional to the $I_{4\times 4}$ matrix because it does not affect the BC. Conversely, the inter-sublattice hopping processes are represented as follows:

$$h_{\mathrm{AB}}(\mathbf{k}) = h^{\dagger}_{\mathrm{BA}}(\mathbf{k}) = \begin{pmatrix} w(\mathbf{k}) & 0 \\ 0 & \gamma w(\mathbf{k}) \end{pmatrix}, \tag{2}$$

where $w(\mathbf{k}) = t_2 + t_2 e^{-i(k_x+k_y)} + t_3 e^{-ik_x} + t_3 e^{-ik_y}$. Here, a dimensionless real number γ is introduced to distinguish hopping processes between different d-orbitals.

By appropriately tuning the model parameters, we can identify four Dirac nodes along $k_y = \pm k_x$. These Dirac points are protected by mirror symmetry with respect to the (11) and $(1-1)$ planes. Notably, the off-diagonal elements of h_{AA} and h_{BB} disappear when $k_y = \pm k_x$ because $v(k_x, \pm k_y) = 0$. In other words, the Hamiltonian H_0 separates into two blocks corresponding to d_{z^2} and $d_{x^2-y^2}$ orbitals, which possess different mirror eigenvalues from each other. As a result, bands from different blocks can intersect each other without gap-opening along $k_y = \pm k_x$, giving rise to Dirac nodes, as illustrated in Fig. 5(b). The four band dispersions along $k_y = \pm k_x$ are stated as follows:

$$\begin{cases} E_{1,\pm}(k) = 2t_0 \cos k \pm \sqrt{\epsilon_0^2 + d_{\mathbf{k}}^2} \\ \\ E_{2,\pm}(k) = 2t_0 \cos k \pm \sqrt{\epsilon_0^2 + \gamma^2 d_{\mathbf{k}}^2}, \end{cases} \tag{3}$$

where $k = \sqrt{k_x^2 + k_y^2}$ and $d_{\mathbf{k}} = t_2 \cos k + t_3$.

We obtained the effective 2×2 linear Hamiltonian around the Dirac points as follows. We denote four Dirac nodes as $\mathbf{k}_0^{(1)} = (k_0, k_0)$, $\mathbf{k}_0^{(2)} = (-k_0, k_0)$, $\mathbf{k}_0^{(3)} = (-k_0, -k_0)$, and $\mathbf{k}_0^{(4)} = (k_0, -k_0)$, where k_0 is a positive number obtained from the band-crossing condition $E_{1,+}(k_0) = E_{2,-}(k_0)$. At each Dirac point, the eigenvectors of the two bands crossing each other are of the form $\mathbf{v}_1^{(j)}(k_0) = (\alpha_1^{(j)}, 0, \beta_1^{(j)}, 0)^{\mathrm{T}}$ and $\mathbf{v}_2^{(j)} = (0, \alpha_2^{(j)}, 0, \beta_2^{(j)})^{\mathrm{T}}$. By projecting the original vector space described by $H_0(\mathbf{k})$ onto the subspace spanned by $\mathbf{v}_1^{(j)}(k_0)$ and $\mathbf{v}_2^{(j)}(k_0)$, we obtain four 2×2 effective Hamiltonians around the four Dirac nodes given by

$$H^{(j)}_{0,\mathrm{eff}}(\mathbf{q}) = f^{(j)}_{0,x}(\mathbf{q})\sigma_x + f^{(j)}_{0,z}(\mathbf{q})\sigma_z, \tag{4}$$

where $\mathbf{q}$ is the momentum with respect to the Dirac point at the j-th quadrant of the BZ and σ_α is the Pauli matrix. Here, $f_{0,x}^{(j)} = C_1(s_{x,j}q_x - s_{y,j}q_y)$ and $f_{0,z}^{(j)} = C_2(s_{x,j}q_x + s_{y,j}q_y)$, where C_1 and C_2 are real-valued coefficients determined by the band parameters, and $s_{x,1} = -s_{x,2} = -s_{x,3} = s_{x,4} = s_{y,1} = s_{y,2} = -s_{y,3} = -s_{y,4} = 1$. As a result, the effective Hamiltonian satisfies $H_{0,\text{eff}}^{(1)}(q_x, q_y) = H_{0,\text{eff}}^{(2)}(-q_x, q_y) = H_{0,\text{eff}}^{(3)}(-q_x, -q_y) = H_{0,\text{eff}}^{(4)}(q_x, -q_y)$, reflecting the mirror symmetry of the original Hamiltonian $H_0(\mathbf{k})$. The corresponding Dirac cones are illustrated in Fig. 5(b).

Next, we consider the SOC between d-orbitals. Note that the matrix elements corresponding to the SOC between the considered d-orbitals, $d_{x^2-y^2}$ and d_{z^2}, vanish at the same site due to the selection rule for the magnetic quantum number. However, when the SOC is introduced, finite hopping processes can occur between $d_{x^2-y^2}$ and d_{z^2} orbitals at different sites. When the SOC is included, these orbitals are mixed with other t_{2g}-orbitals at the same site and modified to

$$|\tilde{d}_{m,\sigma}\rangle \approx |d_{m,\sigma}\rangle + \sum_{n\in t_{2g},\sigma'} c_{m\sigma,n\sigma'}|d_{n,\sigma'}\rangle, \tag{5}$$

as illustrated in Fig. 5(a), where m represents the two e_g-orbitals and $c_{m\sigma,n\sigma'} = \langle d_{n,\sigma'}|H_{\text{SOC}}|d_{m,\sigma}\rangle/(E_{m,\sigma} - E_{n,\sigma'})$. As a result, one can have a nonzero hopping parameter between $d_{x^2-y^2}$ and d_{z^2} orbitals at the neighboring A and B sites. For example, the hopping interaction between $d_{x^2-y^2}$ and d_{z^2} orbitals at $(0,0)$ and $\boldsymbol{\delta}_1 = (a,a)/2$ is given by

$$-u_1 = \langle \tilde{d}_{z^2,\uparrow}(0)|H|\tilde{d}_{x^2-y^2,\uparrow}(\boldsymbol{\delta}_1)\rangle, \tag{6}$$

$$= i\frac{\lambda}{\Delta}\langle d_{z^2,\uparrow}(0)|H_0|d_{xy,\uparrow}(\boldsymbol{\delta}_1)\rangle, \tag{7}$$

where $H = H_0 + H_{\text{SOC}}$ is the full Hamiltonian that includes the SOC, $\lambda = -i\langle d_{xy,\uparrow}|H_{\text{SOC}}|d_{x^2-y^2,\uparrow}\rangle$, and $\Delta = |E_{xy,\uparrow} - E_{x^2-y^2,\uparrow}|$. Considering the half-metallicity of CoS_2, we disregard the spin-flipping terms appearing in Eq. 5, so $H_{\text{SOC}} \propto L_z S_z$. Similarly, we denote the SOC-induced hopping interactions between the A site at $(0,0)$ and B sites at $\boldsymbol{\delta}_2 = (-a,a)/2$, $\boldsymbol{\delta}_3 = (-a,-a)/2$, and $\boldsymbol{\delta}_4 = (a,-a)/2$ as u_2, $-u_1$, and u_2 respectively. This mirrors the inversion symmetry of the system, as depicted in the middle panel of Fig. 5(b). If the mirror symmetry is preserved, $u_1 = u_2$. Using the same projection procedure used to obtain $H_{0,\text{eff}}^{(j)}$, we acquire four 2×2 effective SOC matrices around the four Dirac points, stated as

$$H_{\text{SOC,eff}}^{(j)} = m_{\text{SOC}}^{(j)}\sigma_y, \tag{8}$$

up to the leading order, where

$$m_{\text{SOC}}^{(j)} = C_3\tilde{u}_j(1 - \cos k_0), \tag{9}$$

$$= (-1)^{j-1}C_3\tilde{u}_1(1 - \cos k_0). \tag{10}$$

Here, the coefficient C_3 is determined by the band parameters and $\{\tilde{u}_1, \tilde{u}_2, \tilde{u}_3, \tilde{u}_4\} = \{-u_1, u_2, -u_1, u_2\}$. We overlook the terms of $H_{\text{SOC,eff}}^{(j)}$ linear in q_x and q_y because they don't contribute to the leading terms of the BC as demonstrated in the next paragraph. Note that the SOC gap exhibits a d-wave-like structure, which flips its sign as we change the quadrant in momentum space as shown in the middle panel of Fig. 5(b).

The BC of a general 2×2 Hamiltonian of the form $H(\mathbf{k}) = \sum_{\alpha=x,y,z} h_\alpha(\mathbf{k})\sigma_\alpha$ is given by

$$\Omega_z(\mathbf{k}) = -\frac{\tilde{\mathbf{h}}(\mathbf{k})}{2}\cdot\left(\partial_{k_x}\tilde{\mathbf{h}}(\mathbf{k}) \times \partial_{k_y}\tilde{\mathbf{h}}(\mathbf{k})\right), \tag{11}$$

where

$$\tilde{\mathbf{h}}(\mathbf{k}) = \frac{h_x(\mathbf{k})\hat{x} + h_y(\mathbf{k})\hat{y} + h_z(\mathbf{k})\hat{z}}{\sqrt{h_x(\mathbf{k})^2 + h_y(\mathbf{k})^2 + h_z(\mathbf{k})^2}}, \tag{12}$$

and h_α's are real-valued functions. Applying this formula to our effective Hamiltonian $H^{(j)}_{\text{eff}}(\mathbf{q}) = H^{(j)}_{0,\text{eff}}(\mathbf{q}) + H^{(j)}_{\text{SOC,eff}}(\mathbf{q})$, the leading order of the BC corresponding to the massive Dirac dispersion in the j-th quadrant is obtained as

$$\Omega^{(j)}_z(\mathbf{q}) \approx \frac{m^{(j)}_{\text{SOC}}}{|m^{(j)}_{\text{SOC}}|^3}\Gamma^{(j)}_0(\mathbf{q}), \tag{13}$$

where

$$\Gamma^{(j)}_0(\mathbf{q}) \equiv \frac{\partial f^{(j)}_{0,z}}{\partial q_y}\frac{\partial f^{(j)}_{0,x}}{\partial q_x} - \frac{\partial f^{(j)}_{0,z}}{\partial q_x}\frac{\partial f^{(j)}_{0,x}}{\partial q_y}, \tag{14}$$

$$= 2C_1 C_2 s_{x,j} s_{y,j}, \tag{15}$$

$$= 2(-1)^{j-1} C_1 C_2. \tag{16}$$

Note that the quantity $\Gamma^{(j)}_0(\mathbf{q})$, which is determined by the unperturbed Dirac Hamiltonian $H^{(j)}_{0,\text{eff}}$, has odd symmetry according to the mirror operations with respect to x and y axes, as illustrated in the left panel of Fig. 5(b). This is because the quantities $\partial_{q_x} f^{(0)}_{0,\alpha}$ and $\partial_{q_y} f^{(0)}_{0,\alpha}$ are odd with respect to x and y axes, respectively, while the unperturbed Hamiltonian $H^{(j)}_{0,\text{eff}}(\mathbf{q})$ respects mirror symmetry. Because both $m^{(j)}_{\text{SOC}}$ and $\Gamma^{(j)}_0(\mathbf{q})$ have odd symmetry with respect to the mirror symmetries, BC, which is a product of them, exhibits the same sign over the entire BZ.

7 Magnetic property measurements

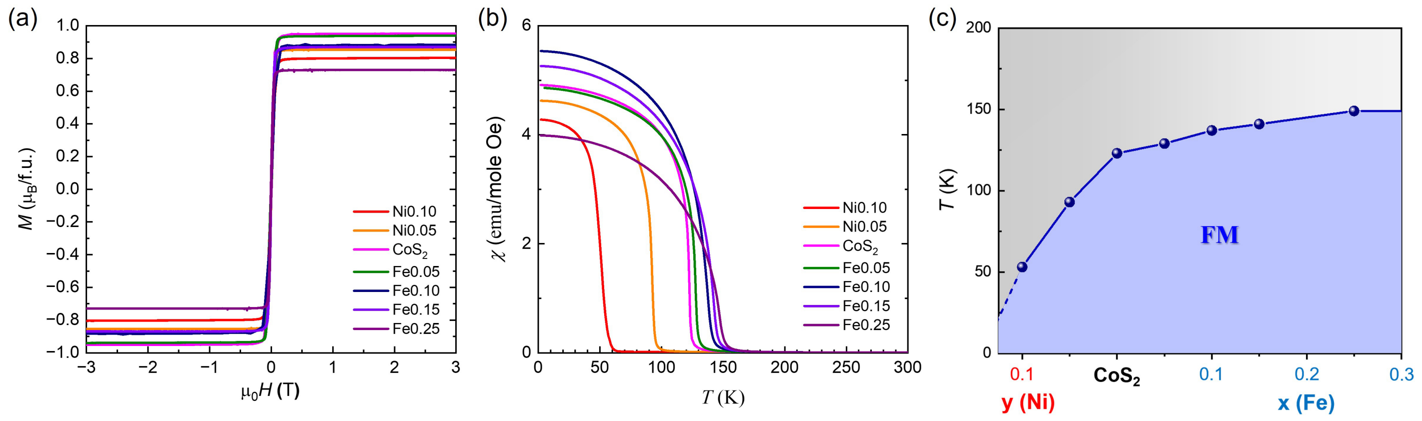

Figure S6. (a) Field dependence of isothermal magnetization measured in $H \parallel a$ at 2 K for $Co_{1-x}Fe_xS_2$ ($x = 0.05$, 0.10, 0.15, and 0.25), CoS_2, and $Co_{1-y}Ni_yS_2$ ($y = 0.05$ and 0.10) single crystals, respectively. (b) Mangetic susceptibility, M/H, measured upon warming at $\mu_0 H = 0.1$ T after zero-field cooling in $H \parallel a$. (c) Phase diagram of Curie temperature T_c. FM denotes the ferromagnetic interaction below T_c.

In order to characterize the magnetic properties of $Co_{1-x}Fe_xS_2$, CoS_2, and $Co_{1-y}Ni_yS_2$ single crystals, field dependence of isothermal magnetization and temperature dependence of magnetic susceptibility, M/H, were measured, as shown in Figure S6(a) and (b), respectively. All the crystals manifest soft magnet behavior, as decribed in Figure S6(a). The saturated moment of CoS_2 is $\sim 0.95\,\mu_B$ in formula unit ($\sim 0.95\,\mu_B$/Co), and those of Fe0.15 and Fe0.25 reach $\sim 1\,\mu_B$/Co, indicative of the half-metallic property. Magnetic susceptibility measured upon warming at $\mu_0 H = 0.1$ T after zero-field colling in $H \parallel a$ exhibits the ferromagnetic interaction obviously (Figure S6(b)). Upon increasing in Fe-doping, Curie temperature T_C increases, as displayed in Figure S6(c).

8 Intrinsic and extrinsic contributions to the anomalous Hall conductivity

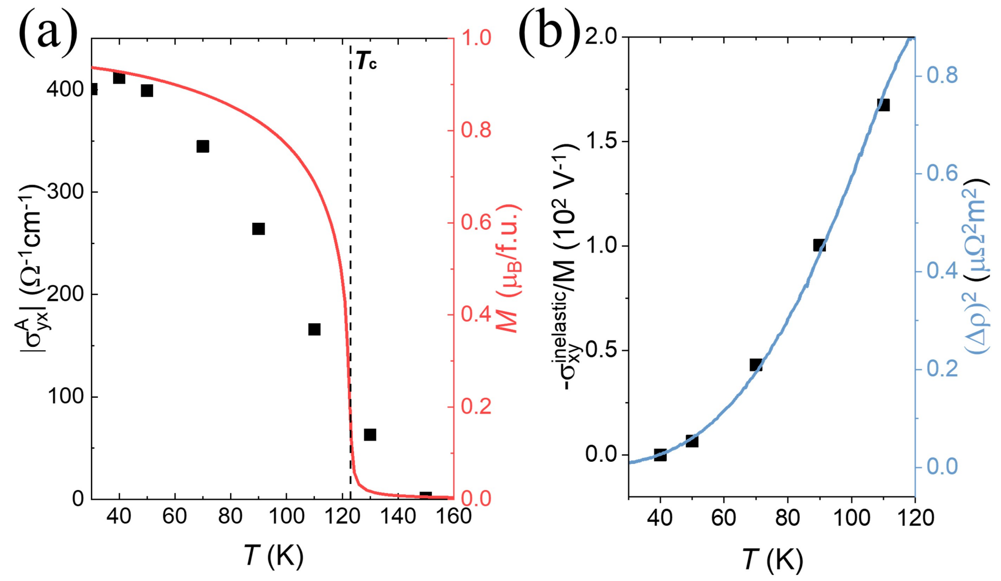

Figure S7. (a) A comparison between the AHC and the magnetization of CoS_2 at temperatures above 30 K. It specifically illustrates how the temperature dependence of AHC correlates with magnetization. (b) Comparison of the inelastic part of Hall conductivity with the square of the inelastic part of the resistivity.

We have expanded our approach beyond the simple log-scale plot presented in Figure 2(a). While the initial data for CoS_2 and Fe0.05 in Figure 2(a) appears almost flat, indicating potential intrinsic behavior within the moderate longitudinal conductivity range, we observed a deviation at higher temperatures. This deviation, characterized by decreasing conductivity on the x-axis, is indicative of an extrinsic mechanism and analyzes it in more detail.

In Figure S7(a), we presented a comparison between the temperature dependence of the AHC and magnetization of CoS_2. The observation that the AHC (black squares) decreases more rapidly than the magnetization (red curve) upon warming up to the T_c indicates that the Hall factor ($S_H = \frac{\sigma^A_{yx}}{M}$) is not constant within this temperature range.

In PNAS 2022, the authors attempted to distinguish between intrinsic and extrinsic components of the AHE using the TYJ model with the relation $\rho^A_{yx} \propto (\rho_{xx})^2$.[1] Both the Karplus-Luttinger model that we analyzed and the TYJ model assume a constant Hall factor.[6] Given this basis, it is reasonable to separate the Hall factor into temperature-independent and temperature-dependent components of the Hall factor.

In Figure S7(b), the temperature-dependent portion of the Hall factor ($\frac{\sigma^{inelastic}_{yx}(T)}{M(T)} = \frac{|\sigma^A_{yx}(T)|}{M(T)} - \frac{\sigma^A_{yx}(40\,\mathrm{K})}{M(40\,\mathrm{K})}$) is plotted by the black squares. This component was derived by subtracting the temperature-independent Hall factor ($\frac{\sigma^A_{yx}(40\,\mathrm{K})}{M(40\,\mathrm{K})}$) from the overall Hall factor ($\frac{|\sigma^A_{yx}(T)|}{M(T)}$), based on the Checkelsky *et al.* (2008).[7] This temperature-dependent Hall factor correlates closely with the square of the inelastic portion of the resistivity (shown by the blue curve, representing $(\Delta\rho(T))^2 = (\rho(T) - \rho(0))^2$). This correlation suggests that electron-magnon scattering and similar inelastic scattering contribute to the decline in the Hall factor.

References

[1] S. Zhang, Y. Wang, Q. Zeng, J. Shen, X. Zheng, J. Yang, Z. Wang, C. Xi, B. Wang, M. Zhou, R. Huang, H. Wei, Y. Yao, S. Wang, S. S. P. Parkin, C. Felser, E. Liu, B. Shen, *Proceedings of the National Academy of Sciences* **2022**, *119* e2208505119.

[2] N. B. M. Schröter, I. Robredo, S. Klemenz, R. J. Kirby, J. A. Krieger, D. Pei, T. Yu, S. Stolz, T. Schmitt, P. Dudin, T. K. Kim, C. Cacho, A. Schnyder, A. Bergara, V. N. Strocov, F. de Juan, M. G. Vergniory, L. M. Schoop, *Science Advances* **2020**, *6* eabd5000.

[3] I. Robredo, N. B. M. Schröter, A. Reyes-Serrato, A. Bergara, F. de Juan, L. M. Schoop, M. G. Vergniory, *Journal of Physics D: Applied Physics* **2022**, *55* 304004.

[4] I. I. Mazin, *Applied Physics Letters* **2000**, *77* 3000.

[5] C.-J. Yu, H. Emmerich, *Journal of Physics: Condensed Matter* **2007**, *19* 306203.

[6] N. Nagaosa, J. Sinova, S. Onoda, A. H. MacDonald, N. P. Ong, *Reviews of Modern Physics* **2010**, *82* 1539.

[7] J. G. Checkelsky, M. Lee, E. Morosan, R. J. Cava, N. P. Ong, *Physical Review B* **2008**, *77* 014433.
